# P‐Wave Parameter Changes After Pulsed‐Field Ablation, Cryoballoon Ablation and Radiofrequency Ablation for Paroxysmal Atrial Fibrillation: An Observational Cohort Study

**DOI:** 10.1002/joa3.70224

**Published:** 2025-11-17

**Authors:** Ibrahim Antoun, Ahmed Abdelrazik, Xin Li, Mahmoud Eldesouky, Kaung Myat Thu, Riyaz Somani, G. André Ng

**Affiliations:** ^1^ Department of Cardiology University Hospitals of Leicester NHS Trust, Glenfield Hospital Leicester UK; ^2^ Department of Cardiovascular Sciences, Clinical Science Wing University of Leicester, Glenfield Hospital Leicester UK; ^3^ Department of Engineering, University of Leicester Leicester UK; ^4^ National Institute for Health Research Leicester Research Biomedical Centre Leicester UK; ^5^ Leicester British Heart Foundation Centre of Research Excellence, Glenfield Hospital UK

**Keywords:** atrial fibrillation, Cryoballoon ablation, pulsed‐field ablation, P‐wave, radiofrequency ablation

## Abstract

**Background:**

Pulmonary vein isolation (PVI) is central to controlling paroxysmal atrial fibrillation (AF). Pulsed‐field ablation (PFA) offers a non‐thermal alternative to conventional thermal techniques. The study compares changes in P‐wave parameters following PFA, radiofrequency ablation (RF), and cryoballoon ablation (Cryo).

**Methods:**

We retrospectively analysed 283 patients undergoing first‐time PVI (RF 101, Cryo 125, PFA 57). Digital electrocardiograms (ECGs) were evaluated for P‐wave duration (PWD), voltage (PWV), dispersion (PWDisp), and terminal force in lead V1 (PTFV1) before and immediately after ablation. AF recurrence was assessed at 12 months. Analyses were adjusted for left atrial volume index (LAVI) and post‐procedural anti‐arrhythmic drug (AAD) use.

**Results:**

At 12 months, 215 patients (76%) remained free of AF (RF 76%, Cryo 74%, PFA 79%; *p* = 0.78). Baseline PWD was 128.5 ± 14 ms (RF), 123.7 ± 15 ms (Cryo), and 124.2 ± 16 ms (PFA). Post‐ablation, mean ΔPWD was +12.2 ms (RF), +8.5 ms (Cryo), and + 4.7 ms (PFA). PTFV1 decreased after all modalities: RF −3.3 to −4.6 mm·ms (*p* < 0.001), Cryo −3.4 to −5.3 mm·ms (*p* = 0.002), PFA −3.6 to −5.2 mm·ms (*p* = 0.005). No significant intergroup differences were observed (*p* = 0.39). Patients with AF recurrence (*n* = 68) had longer baseline PWD (128 ± 16 vs. 125 ± 14 ms, *p* = 0.12), longer post‐procedural PWD (138 ± 17 vs. 129 ± 15 ms, *p* = 0.004), and larger LAVI (29.1 ± 7.9 vs. 25.3 ± 8.5 mL/m^2^,*p* = 0.03). In multivariable Cox models, increased post‐procedural PWD independently predicted recurrence (HR: RF 1.17, Cryo 1.14, PFA 1.13; all *p* < 0.05).

**Conclusions:**

PFA, RF, and Cryo produce similar acute ECG changes. Post‐procedural PWD was the strongest predictor of AF recurrence, independent of atrial size and AAD use.

## Graphical abstract

Pulsed‐field ablation, radiofrequency, and cryoballoon ablation produced similar acute changes in P‐wave indices on surface ECG. Increased post‐procedural P‐wave duration was the only robust predictor of atrial fibrillation recurrence, highlighting its prognostic value across modalities despite the limited sensitivity of body surface ECG for modality‐specific effects.

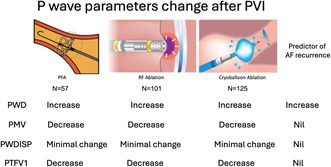

## 1 Introduction

Atrial fibrillation (AF) prevalence and incidence have escalated considerably during the past two decades. They are expected to escalate further, thus constituting one of the most formidable public health threats worldwide [1, 2, 3, 4, 5, 6]. This trend is also observed in the UK, and projections suggest that the AF burden will increase significantly over the next 30 years [7]. Pulmonary vein isolation (PVI) is currently considered the mainstay of rhythm control treatment for symptomatic paroxysmal AF (PAF), especially when pharmacological treatment is ineffective.

The two main energy sources employed for PVI in recent years have been point‐by‐point radiofrequency ablation (RF) and one‐shot cryoballoon ablation (Cryo). RF causes cardiac tissue damage through resistive heating, while Cryo results in tissue death through cryothermal injury. Despite their procedural differences, the two modalities showed comparable results regarding rhythm control in the Fire and Ice trial [8]. Nevertheless, the effects of the two approaches on atrial tissue and electrophysiological remodeling are still quite small and are under investigation.

Pulsed‐field ablation (PFA) is a new treatment modality recently introduced to standard clinical practice. PFA uses non‐thermal irreversible electroporation to destroy myocardial cells. It has the advantage of being highly specific for the tissue and avoiding collateral damage to the esophagus, phrenic nerve, and pulmonary veins. The results of the first clinical trials indicate that PFA has non‐inferior clinical efficacy rates compared to RF and Cryo, with shorter procedural times and fewer adverse events [9, 10]. The main drawback of the current methods is the scarcity of data regarding their electrophysiological impact, especially regarding P‐wave parameters that can be measured from the ECG. The P wave, the first deflection on the ECG, represents atrial depolarisation and has been used as a prognostic marker for recurrence of AF after cardioversion or ablation [11]. Some of the indices that are being used include corrected P‐wave duration (PWD), which represents total time of atrial depolarisation, P‐wave amplitude (PWV) which reflects the electrical strength of atrial depolarisation, P‐wave dispersion (PWDisp) which represents variability in atrial conduction times across different atrial regions, and P‐wave terminal force in lead V1 (PTFV1) which reflects delayed or abnormal activation of the left atrium [12]. These parameters may provide mechanistic information on how different ablation strategies influence atrial electrophysiology. In previous studies, the changes in P‐wave have been evaluated mainly after RF or Cryo [13]. For instance, ΔP‐wave vector magnitude (ΔPvm) has been observed to differ between the two modalities, with Cryo inducing more significant changes than RF, possibly due to the lesion geometry and effects on inter‐atrial conduction pathways. Nevertheless, there is very little data comparing all three modalities regarding P‐wave parameters, namely, RF, Cryo, and PFA.

This study investigates the changes in P‐wave parameters after RF, Cryo, and PFA in patients with PAF undergoing PVI for the first time. Thus, we attempt to define the electrophysiological effects of each technique by measuring PWD, PWV, PWDisp, and PTFV1 before and after ablation and their relation to long‐term procedural success. The information gained from this study may help in risk stratification and procedural selection in the management of PAF.

## 2 Methods

This retrospective observational study examines PAF patients who underwent PVI using PFA between March 2023 and July 2024 at Glenfield Hospital in Leicester, UK. PFA patients were compared to consecutive patients who completed their first PVI for PAF (RF or Cryo) between January 2018 and December 2019. RF was performed with contact force, and second‐generation catheters were employed for Cryo. Patient demographics and medication details were obtained electronically by reviewing clinic appointment letters, which contained clinical information, medications, ablation details, and follow‐up appointments. Patients taking amiodarone before the procedure were excluded, as amiodarone can alter P‐wave morphology. Exclusions also applied to patients with prior ablation procedures, pacing devices, and valvular disease.

Additionally, patients who did not complete their 12‐month follow‐up were excluded. Patients with further ablations outside of PVs were also excluded. Digitized 12‐lead ECG recordings continuously monitored during the procedures were exported for analysis. Patients receiving amiodarone within 3 months before the index procedure were excluded. Class IC agents (flecainide) were withheld for at least five half‐lives (a minimum of 3–5 days), and sotalol was withheld for at least 72 h before the procedure. The digitized ECGs used for P‐wave analysis were acquired immediately before the first energy delivery and immediately after confirmed PVI during the same procedure, before reinitiation of any antiarrhythmic drugs.

Procedure success was defined as the absence of ECG‐documented AF or atrial flutter following the 3‐month blanking period and up to 12 months after ablation, assessed through 12‐lead ECG or ambulatory monitoring. AF recurrence was defined as AF lasting 30 s or more on Holter monitoring (24–72 h). The study was reviewed and ethically approved by the University of Leicester ethical committee (reference number: 35479‐ia196). The reporting of this observational study adhered to the Strengthening the Reporting of Observational Studies in Epidemiology (STROBE) guidelines [14].

### 2.1 Ablation Details

For RF, a circular mapping catheter was deployed following transseptal access in the superior and inferior PVs, followed by circumferential ablation of the left‐sided and right‐sided ipsilateral PVs. PVI was conducted using a 3.5‐mm ablation catheter with an irrigated tip (ThermoCool SmartTouch Catheter, Biosense‐Webster, Diamond Bar, California), guided by the ablation index. Post‐procedure, dormant conduction of the PVs was interrogated using rapid intravenous adenosine injection, and further ablation was performed to achieve PVI.

For the Cryo group, a long sheath (8.5 Fr SL0, Abbott Laboratories or LAMP‐90) was used to obtain transseptal access. The long sheath was then exchanged for a steerable sheath (FlexCath Advance, Medtronic, Minneapolis, MN, USA) to facilitate the positioning of a second‐generation 28 mm cryoballoon (Arctic Front Advance, Medtronic) in the left atrium. An inner‐lumen circumferential mapping catheter (Achieve, Medtronic) was used to assess pulmonary vein (PV) potentials. Cryo was performed at the antrum of the superior and inferior left and right PVs to achieve bidirectional conduction block between the left atrium and PVs. By standard practice, continuous diaphragmatic pacing was performed during ablation of the right‐sided PVs, and energy delivery was terminated if loss of diaphragmatic capture was observed.

PFA procedures were performed using the Farapulse PFA system (Boston Scientific Inc., Menlo Park, CA). Following administration of intravenous heparin, a 12F, 31 mm multielectrode pentaspline PFA catheter (Farawave; Boston Scientific Inc.) was advanced through a 13F deflectable sheath (Faradrive; Boston Scientific Inc.) into the left atrium. The catheter was positioned to ensure circumferential contact of the splines at the PV antrum. Two applications (2.5 s and 2 kV per application) were administered in the “basket” configuration; then, the catheter was slightly rotated (30°–40°) before the delivery of two additional applications. This sequence of ablation was repeated in the “flower” configuration. This protocol was applied at each PV, whilst two further applications were delivered to each of the two superior PVs with anterior torque. Phrenic nerve stimulation was not performed routinely. PVI was assessed as the lack of discrete PV/atrial potential recording at the PV antra, directly using the Farawave catheter and conducting PV stimulation to test for PV exit block.

### 2.2 ECG Tracing and P Wave Measurements

One minute of digital ECG recording (16 bit, voltage range −5 to 5 mV, 1–50 Hz bandpass with notch filter) directly before PVI and 1 min of digital ECG directly after PVI were exported. The ECG data file was exported from LabSystems Pro in the format of .txt files and imported into MATLAB for analysis. The P‐wave peak was detected as the peak with a minimum duration/width of 15 ms in the window of interest for P‐wave peak detection.

**FIGURE 1:**
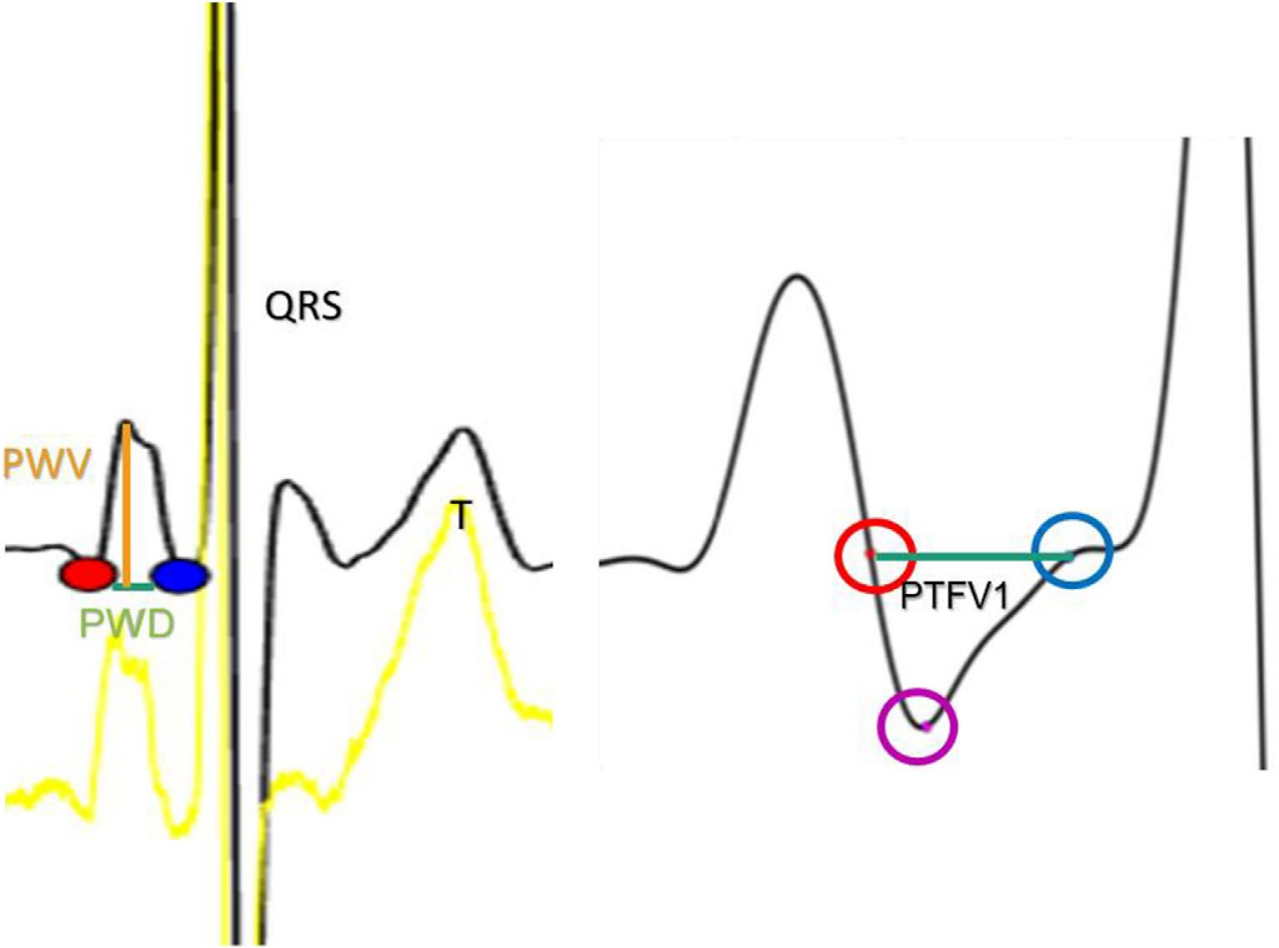
Methodology of P‐wave annotations and measurements. The red dot marks the beginning of the P‐wave, and the blue dot marks the end of the P‐wave. The purple dot marks the most negative point of the P‐wave in V1. PTFV1, P wave terminal force in V1; PWD, P‐wave duration; PWV, P‐wave amplitude.

The P‐wave onset detection window was defined from the T‐wave end to the P‐wave peak. P‐wave onset was detected and defined as the point with the minimum perpendicular distance to the line connecting the two T‐wave and P‐wave peak points. The MatLab script allows interactive operations, allowing users to censor and adjust detected points. Twenty P‐wave measurements were averaged to represent the mean P‐wave parameter in each lead. The P‐wave beginning is defined by the first point of rise above the isoelectric line, while the P‐wave point with the largest vertical distance from the isoelectric line defines the P‐wave peak. These automated detections can be adjusted manually during the measurement process for accurate annotations (Figure 1).

The following P‐wave parameters are produced:

1. PWD: Distance from P‐wave onset to offset.
2. PWV: The area under the P‐wave was estimated using the trapezoidal method, which involves integrating the total area into a little trapezoid.
3. PWDisp: The max difference between P‐wave durations.
4. PTFV1 was defined as the product of the maximum absolute amplitude and duration of the second half of the biphasic P‐wave in mm.ms.

### 2.3 Statistical Analysis

Categorical variables were expressed as frequency and percentage. The mean ± standard error of the mean was adopted to describe continuous parametric data. Pearson's Chi‐squared or Fisher's exact test was used to compare categorical variables between groups. Student's *t*‐tests and Kruskal–Wallis tests were used to compare continuous variables between the groups, depending on the normality of the distribution. Differences in P‐wave parameters across ablation modalities and ECG leads were assessed using a two‐way ANOVA with Bonferroni post hoc corrections. The F‐statistic was calculated with 22 and 1304 degrees of freedom (F_22,1304), representing the between‐groups (leads and ablation modality interaction) and within‐groups (error) terms, respectively. Baseline continuous variables were compared with one‐way ANOVA or Kruskal–Wallis tests, as appropriate, and categorical variables with Chi‐square tests. Univariate comparisons were performed between patients with and without AF recurrence at 12 months using Chi‐square or Fisher's exact tests for categorical variables and Student's *t*‐test or Mann–Whitney *U* test for continuous variables, as appropriate. Variables associated with recurrence at *p* < 0.05 in univariate analysis were entered into multivariable Cox regression models to identify independent predictors. To analyze pre‐ to post‐ablation changes in P‐wave parameters across leads and modalities, we fitted linear mixed‐effects models with random intercepts for patients and for leads nested within patients. Fixed effects included time (pre vs. post), modality (RF, Cryo, PFA), lead, and their interactions. LAVI (mL/m^2^) was included as a covariate. For regression analyses, PWD was scaled in 10‐ms increments to improve clinical interpretability. Sensitivity analyses were performed using 10‐ms increments and increments of one standard deviation. For AF recurrence, multivariable Cox models adjusted for age, sex, LAVI, BMI, hypertension, and post‐procedural AAD use. Inference used Satterthwaite degrees of freedom, and the Benjamini‐Hochberg procedure controlled for multiplicity across 12 leads. Statistical analysis was performed in R (lme4 v1.1‐, lmerTest v3.1‐), Stata 18, and GraphPad Prism v9.3.

### 2.4 Intraobserver Variability Test

There is a human factor in analysing the P‐wave and manually annotating the P‐wave's start and end. Therefore, intraobserver variability tests were conducted to establish the data's reproducibility. Randomly selected twenty‐two 12‐lead ECGs were analysed anonymously on two consecutive days. A total of 5280 P‐waves were analysed and compared twice in 2 days. Variability was calculated using raw numbers and a percentage. Intraobserver variability was 0.03 mV ± 0.001, 13% in PWV, 0.4 ± 0.1 mm.ms, 10% in PTFV1, 4.5 ± 0.3 ms, 4% in PWD and 4.5 ± 0.3 ms, 19% in PWDisp.

## 3 Results

A total of 283 patients were included in the final analysis. The cohort comprised 101 patients in the RF group, 125 in the Cryo group, and 57 in the PFA group. Of these, 215 patients (76%) remained free of AF at 12 months: 77 (76%) in the RF group, 92 (74%) in the Cryo group, and 46 (79%) in the PFA group. Mean recurrence time was 6.5 ± 3.2 months following PVI without a significant difference in ablation modalities. Baseline characteristics are shown in Table 1. Given the established relation between atrial size and conduction, we adjusted all models for LAVI.

**TABLE 1:** The study participants' demographics.

	Total (*n* = 283)	RF (*n* = 101)	Cryo (*n* = 125)	PFA (*n* = 57)	*p* value
Success (%)	215 (76%)	77 (76%)	92 (74%)	46 (79%)	0.78
Recurrence time (months)	6.5 ± 3.2	7.1 ± 3.3	6.4 ± 3.2	6 ± 3	0.09
Male (%)*	185 (65%)	69 (68%)	81 (65%)	35 (61%)	0.78
Age (years)	61 ± 6.5	62.3 ± 7.6	59.7 ± 5.6	59 ± 8.8	0.65
Diabetes mellitus (%)	39 (14%)	17 (17%)	18 (14%)	4 (7%)	0.54
Heart failure (%)	51 (18%)	21 (21%)	23 (18%)	7 (12%)	0.23
Cerebrovascular event (%)	41 (14%)	17 (17%)	22 (18%)	2 (4%)	0.87
Ischemic heart disease (%)	25 (9%)	12 (12%)	11 (10%)	2 (4%)	0.46
Hypertension (%)	113 (40%)	38 (38%)	53 (42%)	22 (39%)	0.11
Indexed left atrial volume (mL/m^2^)*	26.2 ± 8.4	24.9 ± 9.3	27.4 ± 11.2	25 ± 6	0.07
Body mass index (Kg/m^2^)*	30 ± 5.3	29 ± 4.6	31 ± 5.8	29 ± 4.9	0.19
Flecainide after PVI (%)	47 (17%)	15 (15%)	17 (14%)	15 (26%)	0.86
Sotalol after PVI (%)	75 (27%)	23 (23%)	25 (20%)	27 (47%)	0.75
Duration of post procedural AAD therapy (months)	6.7 ± 1.5	6.9 ± 2	6.6 ± 1.7	6 ± 3.7	0.69

*Note:* Pre‐procedural washout: flecainide ≥ 3–5 days; sotalol ≥ 72 h; amiodarone within 3 months‐exclusion. Post‐procedural AAD prescriptions reflect therapy after the index procedure and did not affect acute ECG measurement.

Abbreviations: AF, atrial fibrillation; Cryo, cryoballoon ablation; PVI, pulmonary vein isolation; RF, radiofrequency ablation.

* Data followed a normal distribution and were described as mean ± standard deviation.

### 3.1 P‐Wave Parameter Changes

All four P‐wave parameters were analyzed before and after ablation (Tables 2, 3, 4, 5). Baseline pre‐procedural corrected PWD was 128.5 ± 14 ms in the RF group, 123.7 ± 15 ms in the Cryo group, and 124.2 ± 16 ms in the PFA group. Following ablation, mean ΔPWD across leads was +12.2 ms for RF, +8.5 ms for Cryo, and + 4.7 ms for PFA. The magnitude and direction of change were broadly similar across leads, with no statistically significant difference observed between RF, Cryo, and PFA (Bonferroni‐adjusted *p* values > 0.05 for all leads; F(_22_, 1304) = 0.82, *p* = 0.77) as shown in Table 2. The reported Δ values represent the mean of lead‐specific changes across all 12 leads for each ablation modality. To aid interpretation, we also provide modality‐level averages of pre‐ and post‐ablation values in Table S1.

**TABLE 2:** Corrected P wave duration change following ablation using radiofrequency and cryoballoon ablation.

PWD (ms)	Pre RF (*n* = 101)	Post RF (*n* = 101)	RF Δ	Pre Cryo (*n* = 125)	Post Cryo (*n* = 125)	Cryo Δ	Pre PFA (*n* = 57)	Post PFA (*n* = 57)	PFA Δ	Bonferroni's adjusted P
I	122.1 ± 12	134.3 ± 18	12.2	131.3 ± 15	139.8 ± 18	8.5	130.0 ± 17	134.7 ± 19	4.7	0.53
II	124.4 ± 11	144.4 ± 18	20.0	119.4 ± 15	134.0 ± 21	14.6	123.6 ± 14	139.3 ± 17	15.7	0.73
III	132.6 ± 16	143.5 ± 19	10.9	123.4 ± 15	131.2 ± 25	7.8	122.7 ± 17	128.7 ± 16	6.0	0.6
AVR	124.8 ± 9	138.8 ± 22	14.0	129.0 ± 15	138.4 ± 22	9.4	122.7 ± 14	138.0 ± 18	15.3	0.65
AVL	126.3 ± 22	132.9 ± 26	6.6	113.7 ± 15	118.1 ± 26	4.4	140.8 ± 15	146.1 ± 24	5.3	0.45
AVF	137.0 ± 16	145.0 ± 27	8.0	119.3 ± 15	127.8 ± 21	8.5	132.7 ± 14	143.0 ± 23	10.3	0.34
V1	144.3 ± 19	152.0 ± 23	7.7	134.7 ± 15	144.4 ± 17	9.7	119.2 ± 14	134.4 ± 22	15.2	0.58
V2	128.2 ± 11	142.5 ± 19	14.3	122.2 ± 15	132.1 ± 32	9.9	119.7 ± 10	121.8 ± 17	2.1	0.59
V3	131.0 ± 17	139.2 ± 26	8.2	128.0 ± 15	138.3 ± 25	10.3	127.4 ± 18	132.2 ± 22	4.8	0.71
V4	132.6 ± 20	142.4 ± 22	9.8	119.4 ± 15	128.5 ± 19	9.1	105.9 ± 10	119.7 ± 18	13.8	0.64
V5	134.7 ± 30	139.8 ± 25	5.1	122.0 ± 15	130.7 ± 28	8.7	100.6 ± 12	117.5 ± 24	16.9	0.38
V6	134.3 ± 18	140.0 ± 28	5.7	123.8 ± 15	132.0 ± 26	8.2	131.0 ± 16	141.5 ± 18	10.5	0.4

*Note:* Values represent lead‐specific means ± SD. Δ represents mean change within each lead. Reported Δ in the text represents the mean of all 12 lead‐specific Δ values.

Abbreviations: Cryo, cryoballoon ablation; PFA, pulsed‐field ablation; RF, radiofrequency ablation.

**TABLE 3:** P wave voltage change following ablation using radiofrequency and cryoballoon ablation.

Lead	Pre RF (*n* = 101)	Post RF (*n* = 101)	RF Δ	Pre Cryo (*n* = 125)	Post Cryo (*n* = 125)	Cryo Δ	Pre PFA (*n* = 57)	Post PFA (*n* = 57)	PFA Δ	Bonferroni's adjusted P
I	0.13 ± 0.05	0.09 ± 0.04	−0.04	0.14 ± 0.05	0.12 ± 0.04	−0.02	0.07 ± 0.05	0.05 ± 0.07	−0.02	0.57
II	0.24 ± 0.02	0.18 ± 0.07	−0.06	0.23 ± 0.09	0.21 ± 0.04	−0.04	0.21 ± 0.04	0.18 ± 0.03	−0.03	0.87
III	0.15 ± 0.06	0.13 ± 0.06	−0.02	0.19 ± 0.07	0.16 ± 0.04	0.01	0.14 ± 0.03	0.16 ± 0.07	0.02	0.82
|AVR|	0.16 ± 0.06	0.12 ± 0.06	−0.04	0.20 ± 0.07	0.14 ± 0.03	0.02	0.18 ± 0.07	0.01 ± 0.06	−0.17	0.43
AVL	0.07 ± 0.08	0.06 ± 0.03	−0.01	0.08 ± 0.06	0.04 ± 0.02	0.03	0.17 ± 0.03	0.09 ± 0.04	−0.08	0.68
AVF	0.20 ± 0.07	0.17 ± 0.06	−0.03	0.20 ± 0.1	0.15 ± 0.03	0.02	0.08 ± 0.05	0.12 ± 0.06	0.04	0.66
V1	0.12 ± 0.09	0.08 ± 0.04	−0.04	0.09 ± 0.05	0.06 ± 0.01	−0.01	0.16 ± 0.05	0.09 ± 0.06	−0.07	0.37
V2	0.16 ± 0.08	0.20 ± 0.07	0.04	0.14 ± 0.07	0.11 ± 0.03	0.07	0.11 ± 0.06	0.14 ± 0.03	0.03	0.42
V3	0.18 ± 0.07	0.12 ± 0.06	−0.06	0.17 ± 0.08	0.13 ± 0.02	−0.02	0.16 ± 0.04	0.13 ± 0.04	−0.03	0.58
V4	0.16 ± 0.05	0.12 ± 0.04	−0.04	0.19 ± 0.09	0.14 ± 0.03	0.01	0.08 ± 0.05	0.06 ± 0.05	−0.02	0.44
V5	0.15 ± 0.07	0.13 ± 0.06	−0.02	0.16 ± 0.06	0.11 ± 0.04	0.03	0.12 ± 0.06	0.09 ± 0.05	−0.03	0.44
V6	0.13 ± 0.05	0.10 ± 0.05	−0.03	0.16 ± 0.05	0.11 ± 0.04	0.02	0.17 ± 0.05	0.15 ± 0.04	−0.02	0.85

*Note:* Values represent lead‐specific means ± SD. Δ represents mean change within each lead. Reported Δ in the text represents the mean of all 12 lead‐specific Δ values.

Abbreviations: Cryo, cryoballoon ablation; PFA, pulsed‐field ablation; RF, radiofrequency ablation.

PWV showed a consistent decrease following RF and Cryo, ranging from −0.01 mV to −0.06 mV. PFA was associated with variable effects across leads, including mild increases and decreases in amplitude. For instance, leads I and II showed minor reductions (−0.02 to −0.03 mV), while AVR exhibited a larger drop (−0.17 mV). However, the groups showed no statistically significant differences (F (_22_, 1304) = 0.82, *p* = 0.77).

PWDisp demonstrated heterogeneous responses. In RF and Cryo groups, changes ranged from −5.9 ms to +2.7 ms and − 1.5 ms to +4.2 ms, respectively. PFA showed a mix of minimal increases and decreases, ranging from −2.8 ms to +4.6 ms across leads (Table 4). Overall, no significant differences existed between groups in any individual lead (Bonferroni‐adjusted *p* values all > 0.05), although numerically greater variability was observed in the PFA group (F_22_, 1304 = 0.18, *p* = 0.59).

**TABLE 4:** P wave dispersion change following ablation using radiofrequency and cryoballoon ablation.

Lead	Pre RF (*n* = 101)	Post RF (*n* = 101)	RF Δ	Pre Cryo (*n* = 125)	Post Cryo (*n* = 125)	Cryo Δ	Pre PFA (*n* = 57)	Post PFA (*n* = 57)	PFA Δ	Bonferroni's adjusted P
I	28 ± 2.3	30.5 ± 2.7	2.7	32.8 ± 2.8	38.2 ± 2.7	4.2	37.2 ± 2.5	38.1 ± 2.2	1.9	0.72
II	29.7 ± 2.5	28.1 ± 2.6	0.5	31.4 ± 2.7	36.6 ± 2.9	3.9	37.2 ± 2.1	41.6 ± 2.1	3.5	0.65
III	32.8 ± 2.5	32.4 ± 2.8	0.3	35 ± 2.9	40.4 ± 2.6	3.2	31.5 ± 2.2	29.7 ± 2.3	−0.4	0.79
AVR	34.8 ± 2.4	31.4 ± 2.7	−2.4	34.5 ± 4.5	32 ± 6.5	−1.9	35.8 ± 2.0	37.6 ± 2.7	0.2	0.66
AVL	39.8 ± 3.2	31.4 ± 5.3	−5.9	35.7 ± 3.1	36.1 ± 3.1	−1.5	38.1 ± 2.0	35.9 ± 2.1	−2.8	0.6
AVF	32.1 ± 2.8	33.4 ± 2.9	1.8	32.9 ± 2.9	36.4 ± 3.2	2.9	35.6 ± 2.5	37.7 ± 2.2	2.2	0.27
V1	34.7 ± 3	31.4 ± 3	−2.3	35.7 ± 2.8	35.2 ± 2.7	−0.9	34.9 ± 2.2	34.7 ± 2.0	−0.8	0.46
V2	32.8 ± 2.7	31.1 ± 2.4	0.2	34.5 ± 3	35.2 ± 2.5	1.4	29.3 ± 2.7	36.5 ± 2.1	4.6	0.39
V3	33.9 ± 2.7	33.5 ± 3	−0.2	33 ± 2.5	33.5 ± 2.5	0.2	34.9 ± 2.1	34.5 ± 2.7	−0.2	0.37
V4	29.2 ± 2.5	28.4 ± 2.6	−0.5	33.7 ± 2.5	36.1 ± 3.2	1.1	32.5 ± 2.3	30.3 ± 2.7	−1.2	0.88
V5	29.6 ± 2.3	29.9 ± 2.8	1.0	35.2 ± 2.8	35.4 ± 2.2	0.9	31.9 ± 2.7	36.2 ± 2.2	2.9	0.48
V6	34 ± 2.3	35.5 ± 2.8	1.1	33 ± 2.9	37.4 ± 2.7	2.6	34.7 ± 2.8	31.1 ± 2.3	−1.4	0.82

*Note:* Values represent lead‐specific means ± SD. Δ represents mean change within each lead. Reported Δ in the text represents the mean of all 12 lead‐specific Δ values.

Abbreviations: Cryo, cryoballoon ablation; PFA, pulsed‐field ablation; RF, radiofrequency ablation.

**TABLE 5:** Comparison of baseline and procedural characteristics between patients with and without AF recurrence at 12 months.

Characteristic	No recurrence (*n* = 215)	Recurrence (*n* = 68)	*p* value
Age (years)	61.2 ± 6.3	62.0 ± 6.7	0.41
Male (%)	139 (65)	46 (68)	0.66
BMI (kg/m^2^)	29.9 ± 5.4	30.5 ± 5.3	0.42
Hypertension (%)	84 (39)	29 (43)	0.61
Diabetes mellitus (%)	28 (13)	11 (16)	0.53
Ischemic heart disease (%)	19 (9)	6 (9)	0.97
Cerebrovascular disease (%)	28 (13)	13 (19)	0.21
Indexed LA volume (mL/m^2^)	25.3 ± 8.5	29.1 ± 7.9	0.03
Baseline PWD (ms)	125 ± 14	128 ± 16	0.12
Post‐procedural PWD (ms)	129 ± 15	138 ± 17	0.004
ΔPWD (ms)	+8.1 ± 5.2	+10.3 ± 6.1	0.04
Baseline PWV (mV·ms)	0.16 ± 0.06	0.15 ± 0.05	0.29
Baseline PWDisp (ms)	32.5 ± 4.1	33.4 ± 4.3	0.31
Baseline PTFV1 (mm·ms)	−3.4 ± 0.8	−3.5 ± 0.7	0.57
Post‐procedural AAD use (%)	86 (40)	32 (47)	0.29

*Note:* Data are mean ± SD or *n* (%). *p* values from Student's *t*‐test or Chi‐square/Fisher's exact test, as appropriate.

Abbreviations: AAD, anti‐arrhythmic drug; LA, left atrial; PTFV1, P‐wave terminal force in V1; PWD, corrected P‐wave duration; PWDisp, P‐wave dispersion; PWV, P‐wave voltage.

PTFV1 decreased after all three modalities. RF ablation resulted in a reduction from −3.3 mm·ms to −4.6 mm·ms (*p* < 0.001), and Cryo from −3.4 mm·ms to −5.3 mm·ms (*p* = 0.002). PFA showed a comparable decrease (from −3.6 mm·ms to −5.2 mm·ms, *p* = 0.005). The extent of PTFV1 reduction was similar across modalities (*p* = 0.39), suggesting a uniform impact on atrial posterior conduction. Adjustment did not change the direction or significance of time effects on PWD and PTFV1, nor the lack of a modality‐by‐time interaction. Similarly, the association between longer post‐ablation PWD and AF recurrence remained significant after adjustment for LAVI and other covariates. In supplementary analyses adjusting for baseline indexed left atrial volume (LAVI), the magnitude of post‐ablation increases in PWD and reductions in PTFV1 remained significant and did not differ by ablation modality. For AF recurrence, multivariable Cox models including age, sex, BMI, hypertension, LAVI, and post‐procedural AAD use confirmed that longer post‐ablation PWD was independently associated with recurrence across all modalities (RF HR 1.17 per 10 ms, Cryo HR 1.14 per 10 ms, PFA HR 1.13 per 10 ms; all *p* < 0.05). These results are detailed in Table S2.

### 3.2 Predictors of Ablation Failure

Patients who experienced AF recurrence at 12 months had significantly longer baseline PWD (129 ± 15 vs. 124 ± 14 ms, *p* = 0.02), longer post‐procedural PWD (138 ± 17 vs. 129 ± 15 ms, *p* = 0.004), and larger indexed LA volume (29.1 ± 7.9 vs. 25.3 ± 8.5 mL/m^2^, *p* = 0.03) compared with patients who remained arrhythmia‐free. Other baseline clinical factors (age, sex, BMI, hypertension, diabetes, ischemic heart disease, cerebrovascular disease) and P‐wave indices (PWV, PWDisp, PTFV1) did not differ significantly between groups. On univariate Cox regression (Table S2), baseline PWD (per 10 ms, HR 1.12, 95% CI 1.02–1.23, *p* = 0.02), post‐procedural PWD (per 10 ms, HR 1.22, 95% CI 1.07–1.38, *p* = 0.004), and LAVI (per 5 mL/m^2^, HR 1.18, 95% CI 1.02–1.37, *p* = 0.03) were associated with AF recurrence. In multivariable models that included these variables alongside prespecified covariates (age, sex, BMI, hypertension, and post‐procedural AAD use), only post‐procedural PWD remained independently predictive (HR 1.17 per 10 ms, 95% CI 1.04–1.33, *p* = 0.01).

Adjustment for baseline left atrial volume index (LAVI) in mixed‐effects models did not change the direction or significance of the time effects on PWD and PTFV1, and there was no modality‐by‐time interaction. In multivariable Cox models that included age, sex, BMI, hypertension, LAVI, and post‐procedural anti‐arrhythmic drug use, post‐procedural PWD remained independently associated with AF recurrence across modalities (RF HR 1.17 per 10 ms, 95% CI 1.04–1.33; Cryo HR 1.14 per 10 ms, 95% CI 1.02–1.29; PFA HR 1.13 per 10 ms, 95% CI 1.00–1.30; all *p* < 0.05). Full coefficients for the adjusted mixed‐effects and Cox models are provided in Table S3.

## 4 Discussion

This is the first study to compare changes in P wave parameters after PFA to those after RF and cryo. This observational study provides novel insights into the electrophysiological effects of PFA compared to RF and Cryo in patients undergoing first‐time PVI for PAF. This study demonstrates that PFA produces acute changes in P‐wave indices comparable to RF and cryo. PWD increased and PTFV1 decreased across all modalities, without significant intergroup differences. Importantly, PWD was a consistent predictor of AF recurrence, independent of ablation modality. These findings suggest that surface ECG parameters reflect shared electrophysiological consequences of PVI, rather than modality‐specific effects. Although preclinical data indicate that PFA may preserve atrial structure and autonomic innervation, our study did not demonstrate differences in ECG indices to support this finding. Future mechanistic work with imaging and high‐density mapping is required to clarify whether PFA confers distinct long‐term remodeling advantages. However, no significant differences were observed between PFA, RF, and Cryo regarding the magnitude of these changes, suggesting comparable acute impacts on atrial conduction. Our results contrast with the expectation that PFA would produce minimal perturbation of P‐wave parameters [15]. Although previous preclinical and early clinical studies suggested that PFA might preserve atrial conduction more effectively than thermal energy [16], our findings demonstrate that PFA, while tissue‐selective, still induces detectable alterations in atrial depolarisation patterns. Notably, PFA patients exhibited numerically larger increases in PWD in some leads (V5, AVR), although these differences were not statistically significant. Similarly, reductions in PWV and PTFV1 were seen across all modalities.

The biophysical properties of PFA likely underpin its distinct tissue effects. Unlike RF and Cryo, which cause thermal injury with coagulative necrosis and fibrosis, PFA induces non‐thermal, irreversible electroporation of cardiomyocytes while preserving the extracellular matrix and minimising inflammatory responses [17, 18]. This unique healing response produces sharply demarcated lesions with reduced fibrosis and preserved mechanical properties. While acute electrophysiological effects measured on the surface ECG may appear similar to RF and Cryo, the longer‐term consequences of this distinct lesion formation could be highly advantageous in preserving atrial compliance, minimising arrhythmogenic scar formation, and maintaining atrial contractile function.

Interestingly, despite achieving effective PVI, PFA did not significantly disrupt atrial conduction more than thermal methods. This suggests that PFA isolates pulmonary veins primarily by ablating peri‐venous myocardial fibers without causing widespread collateral injury to the surrounding atrial tissue [19]. Emerging clinical data now indicate that despite being non‐thermal, PFA creates larger antral lesion sets than CB ablation. In the study by Blockhaus et al., PFA demonstrated significantly greater antral lesion coverage compared to CB as assessed by high‐density electroanatomical mapping. This finding indicates that PFA can achieve durable PVI and extensive antral debulking without the collateral thermal damage typically associated with RF or Cryo [20]. Importantly, although PFA lesions are larger acutely, they result from myocardial‐specific electroporation rather than widespread coagulative necrosis. The preservation of extracellular architecture and avoidance of excessive fibrosis characteristic of PFA might explain why acute increases in PWD and reductions in PWV and PTFV1 were modest and comparable to thermal methods in our study. Therefore, PFA can achieve broad and effective electrical isolation while minimising long‐term fibrotic remodeling, a feature that distinguishes it mechanistically from thermal ablation strategies. The higher baseline LAVI observed in the PFA cohort could, in principle, predispose to longer conduction times. However, after adjusting for LAVI in mixed‐effects and Cox models, the principal findings were unchanged, suggesting that baseline LA size differences do not drive the observed P‐wave changes and the prognostic value of PWD.

Another consideration is the effect of ablation modality on the cardiac autonomic nervous system. RF and Cryo often incidentally ablate ganglionated plexi (GP) in the peri‐PV region, leading to acute vagal withdrawal and potential autonomic remodeling. PFA's selective action on myocardial cells may spare the GP and surrounding neuronal structures [15], possibly preserving autonomic tone and reducing the risk of post‐procedural autonomic dysfunction. In our cohort, the absence of a heart rate rise post‐PFA suggests that autonomic innervation may have been better preserved than in thermal ablations [21]. However, whether this preservation impacts long‐term rhythm outcomes remains an open question and warrants further investigation.

Given these findings, the role of P‐wave parameters as procedural success markers after PFA deserves reconsideration. Traditional ECG markers such as PWD shortening or PTFV1 reduction have been used to infer effective debulking or atrial remodeling after thermal ablation. In the context of PFA, however, minimal changes in surface ECG indices may not indicate procedural failure but rather reflect targeted, tissue‐sparing ablation. Clinicians should be cautious in overinterpreting the absence of dramatic ECG changes after PFA and should consider complementary methods such as voltage mapping or imaging to assess lesion efficacy. As PFA becomes integrated into clinical practice, the interpretation of P‐wave indices will evolve. These indices remain crucial for understanding a patient's atrial substrate and risk. Still, their post‐procedural changes (or lack thereof) must be viewed through PFA's tissue‐selective effects. Ultimately, our data support that PFA maintains sinus rhythm not by creating more scar than thermal ablation methods, but by precisely eliminating triggers while sparing the rest of the atrium. This approach may usher in improved long‐term efficacy and safety in PVI. An important observation in our study is the consistent association between increased PWD and AF recurrence across all modalities, which agrees with other studies using RF and Cryo [22, 23, 24].

In contrast, PWV, PWDisp, and PTFV1 changes were not independently predictive. This result is notable, as prior studies have reported correlations between abnormal PTFV1 and ablation outcome, likely reflecting thermal lesion effects on atrial tissue [25]. Our findings suggest that while thermal techniques may impact these parameters by inducing fibrosis and altering atrial conduction pathways, PFA's mechanism, targeting myocytes without extensive fibrotic remodeling, preserves atrial architecture, limiting the predictive value of PWV and PTFV1 post‐procedure. Therefore, PWD remains the most robust and universal marker of atrial remodeling and conduction slowing, even in non‐thermal ablation. An important consideration is the timing of our measurements. P‐wave parameters were analysed immediately after PVI, when acute tissue injury and inflammatory responses are at their peak. Even with PFA, acute oedema and transient conduction slowing are likely, and thus the ECG changes we report may not reflect long‐term remodeling. Echocardiographic and cardiac MRI studies have demonstrated that conduction and structural alterations observed acutely after ablation can partially or fully resolve within weeks to months. Therefore, it remains possible that PFA may demonstrate greater reversibility of P‐wave indices over time compared with thermal modalities. Longitudinal assessments with serial ECG and imaging are necessary to determine whether the maturation of PFA lesions results in distinct long‐term electrophysiological profiles. Another important consideration is the inherent limitation of surface ECG for detecting subtle modality‐specific differences. While PWD and PTFV1 consistently changed across modalities, the absence of significant intergroup differences may reflect the restricted sensitivity of body surface recordings rather than true electrophysiological equivalence. More granular tools, such as high‐density electroanatomical mapping, atrial strain imaging, or late gadolinium enhancement cardiac MRI, are better suited to delineate lesion characteristics, atrial remodeling trajectories, and autonomic effects unique to each modality. Integration of ECG with these complementary approaches will be crucial to understand how PFA compares mechanistically with thermal ablation fully.

Future research should focus on validating non‐invasive markers of procedural success specific to PFA. High‐density electroanatomical mapping, atrial strain imaging, and late gadolinium enhancement MRI may provide better insights into the structural effects of PFA. Long‐term studies are also needed to determine whether the apparent preservation of atrial conduction and autonomic function translates into lower rates of atrial arrhythmias, atrial mechanical dysfunction, or stroke.

## 5 Limitations

This study has several limitations. First, it was a single‐centre, retrospective analysis, which may introduce selection bias. Second, atrial fibrillation recurrence was assessed using scheduled ECGs or ambulatory monitoring, without continuous surveillance by implantable loop recorders, and therefore subclinical episodes may have been missed. Third, the sample size was modest and not based on an a priori power calculation, increasing the risk of type II error when comparing ablation modalities. Fourth, the temporal gap between RF/Cryo procedures (2018–2019) and PFA procedures (2023–2024) introduces potential systematic bias, as practice patterns, technology, and peri‐procedural management may have evolved. Although follow‐up schedules, monitoring strategies, and ECG acquisition parameters were consistent across cohorts, residual bias cannot be excluded. Fifth, a greater proportion of patients in the PFA group were discharged on sotalol compared with the thermal groups, which may have confounded recurrence outcomes. To mitigate this, we performed a sensitivity analysis incorporating anti‐arrhythmic drug use as a covariate in the Cox regression model, where increased PWD remained independently predictive of AF recurrence. Sixth, P‐wave analysis was limited to four indices (PWD, PWV, PWDisp, PTFV1) and did not include other morphological or vectorial parameters that might provide additional insights. Finally, only acute pre‐ and post‐ablation ECGs were analyzed, and no longitudinal follow‐up of P‐wave indices was undertaken; such data could help clarify differences in atrial remodeling between thermal and non‐thermal ablation modalities. Because our analysis was confined to ECGs acquired immediately before and after ablation, our findings reflect only the acute electrophysiological response to PVI. Chronic atrial remodeling processes, including lesion maturation, fibrosis, and potential recovery of conduction, may diverge substantially between thermal and non‐thermal modalities. Without longitudinal ECG follow‐up, we cannot determine whether the acute similarity in P‐wave changes between PFA and thermal ablation persists or evolves into distinct patterns over time. This absence of chronic ECG data constrains the interpretation of our results and represents a key area for future research.

## 6 Conclusion

PFA produces acute changes in atrial depolarisation indices similar to those observed after radiofrequency and Cryo. Increased PWD was a robust predictor of AF recurrence across modalities, independent of post‐procedural anti‐arrhythmic therapy. While PFA may offer procedural and safety advantages, our study does not demonstrate modality‐specific differences in surface ECG parameters. Larger prospective studies with longitudinal follow‐up are required to establish whether PFA confers unique electrophysiological or clinical benefits compared with thermal ablation.

## Author Contributions

I.A.: conceptualisation, methodology, validation, formal analysis and data collection. X.L.: validation, software, data curation. A.A., M.E., G.A.N., R.S. and K.M.T.: writing – review and editing. G.A.N.: supervision, writing – review and editing.

## Ethics Statement

The University of Leicester's ethical committee reviewed and ethically approved the study (reference number: 35479‐ia196).

## Conflicts of Interest

The authors declare no conflicts of interest.

## Supporting information


**Table S1:** Overall averaged pre‐ and post‐ablation P‐wave parameters by ablation modality.
**Table S2:** Univariate and multivariable Cox regression analyses for predictors of AF recurrence at 12 months.
**Table S3:** Sensitivity analyses adjusting for baseline left atrial volume index (LAVI) and post‐procedural anti‐arrhythmic drug (AAD) use.
