# Supplementary material for: P‐Wave Parameter Changes After Pulsed‐Field Ablation, Cryoballoon Ablation and Radiofrequency Ablation for Paroxysmal Atrial Fibrillation: An Observational Cohort Study
Source: J Arrhythm. 2025 Nov 17;41(6):e70224. doi: 10.1002/joa3.70224 (PMC12620618; doi:10.1002/joa3.70224)
Supplement: Supplementary file 1 — Table S1: Overall averaged pre‐ and post‐ablation P‐wave parameters by ablation modality. Table S2: Univariate and multivariable Cox regression analyses for predictors of AF recurrence at 12 months. Table S3: Sensitivity analyses adjusting for baseline left atrial volume index (LAVI) and post‐procedural anti‐arrhythmic drug (AAD) use. [file JOA3-41-e70224-s001.docx]

Supplementary table S1: Overall averaged pre- and post-ablation P-wave parameters by ablation modality

| **Parameter** | **RF (n=101)** | **Cryo (n=125)** | **PFA (n=57)** | **P value (ANOVA/KW)** |
| --- | --- | --- | --- | --- |
| Pre PWD (ms) | 128.5 ± 14 | 123.7 ± 15 | 124.2 ± 16 | 0.21 |
| Post PWD (ms) | 138.3 ± 18 | 132.2 ± 19 | 128.9 ± 18 | 0.18 |
| Pre PWV (mV·ms) | 0.16 ± 0.05 | 0.17 ± 0.06 | 0.15 ± 0.05 | 0.34 |
| Post PWV (mV·ms) | 0.12 ± 0.04 | 0.14 ± 0.05 | 0.13 ± 0.06 | 0.41 |
| Pre PWDisp (ms) | 32.1 ± 4.2 | 33.0 ± 3.8 | 34.0 ± 4.0 | 0.27 |
| Post PWDisp (ms) | 33.5 ± 4.1 | 35.1 ± 3.9 | 35.0 ± 3.7 | 0.30 |
| Pre PTFV1 (mm·ms) | −3.3 ± 0.7 | −3.4 ± 0.8 | −3.6 ± 0.9 | 0.19 |
| Post PTFV1 (mm·ms) | −4.6 ± 0.8 | −5.3 ± 0.9 | −5.2 ± 0.8 | 0.23 |

Values represent mean ± SD across all 12 ECG leads, averaged within each patient and then across patients within each modality. Corrected P-wave duration (PWD) is reported in ms, P-wave voltage (PWV) as integrated area (mV·ms), P-wave dispersion (PWDisp) in ms, and P-wave terminal force in V1 (PTFV1) in mm·ms (negative values indicate greater terminal negativity). P values correspond to one-way ANOVA or Kruskal–Wallis tests comparing modalities at each time point.

Lead-specific values are presented in Tables 2–4 of the main manuscript.

Reported Δ values in the Results text reflect the mean of lead-specific changes across all 12 leads within each modality.

This table provides overall modality-level summaries of pre- and post-ablation values to facilitate interpretation.

RF, radiofrequency ablation; Cryo, cryoballoon ablation; PFA, pulsed-field ablation; PWD, corrected P-wave duration; PWV, P-wave voltage; PWDisp, P-wave dispersion; PTFV1, P-wave terminal force in lead V1; LAVI, left atrial volume index; SD, standard deviation; ANOVA, analysis of variance; KW, Kruskal–Wallis test.

Supplementary table S2: Univariate and multivariable Cox regression analyses for predictors of AF recurrence at 12 months

| **Predictor** | **Univariate HR (95% CI)** | **P value** | **Multivariable HR (95% CI)** | **P value** |
| --- | --- | --- | --- | --- |
| Age (per year) | 1.02 (0.98–1.06) | 0.34 | 1.01 (0.97–1.05) | 0.55 |
| Male sex | 1.08 (0.72–1.62) | 0.71 | 1.04 (0.68–1.61) | 0.84 |
| BMI (per kg/m²) | 1.01 (0.96–1.06) | 0.68 | 1.00 (0.95–1.05) | 0.92 |
| Hypertension | 1.14 (0.74–1.75) | 0.56 | 1.11 (0.69–1.78) | 0.65 |
| Diabetes mellitus | 1.21 (0.65–2.23) | 0.55 | – | – |
| Indexed LA volume (per 5 mL/m²) | 1.18 (1.02–1.37) | 0.03 | 1.09 (0.93–1.28) | 0.28 |
| PWV (per 0.1 mV·ms) | 1.05 (0.89–1.24) | 0.55 | – | – |
| Baseline PWD (per 10 ms) | 1.12 (1.02–1.23) | 0.02 | 1.06 (0.95–1.19) | 0.28 |
| Post-procedural PWD (per 10 ms) | 1.22 (1.07–1.38) | 0.004 | 1.17 (1.04–1.33) | 0.01 |
| PWDisp (per 1 ms) | 1.02 (0.97–1.08) | 0.34 | – | – |
| PTFV1 (per 1 mm·ms) | 1.04 (0.91–1.19) | 0.58 | – | – |
| Post-procedural AAD use | 1.24 (0.81–1.90) | 0.31 | 1.19 (0.75–1.88) | 0.46 |

Variables with p < 0.50 in univariate analyses (LAVI, baseline PWD, post-procedural PWD) were entered into the multivariable model, along with prespecified clinical covariates (age, sex, BMI, hypertension, and post-procedural AAD use). Only post-procedural PWD remained independently associated with AF recurrence. HR: hazard ratio.

AF, atrial fibrillation; HR, hazard ratio; CI, confidence interval; RF, radiofrequency ablation; Cryo, cryoballoon ablation; PFA, pulsed-field ablation; PWD, corrected P-wave duration; PWV, P-wave voltage; PWDisp, P-wave dispersion; PTFV1, P-wave terminal force in lead V1; LAVI, left atrial volume index; AAD, anti-arrhythmic drug; BMI, body mass index.

**Supplementary Table S3:** Sensitivity analyses adjusting for baseline left atrial volume index (LAVI) and post-procedural anti-arrhythmic drug (AAD) use

| **Outcome** | **Model** | **Effect estimate** | **95% CI** | **P value** |
| --- | --- | --- | --- | --- |
| **Δ PWD (ms, post–pre)** | Mixed-effects, unadjusted | 9.8 | 8.7 – 10.9 | <0.001 |
|  | Mixed-effects, adjusted for LAVI | 9.6 | 8.5 – 10.8 | <0.001 |
| **Δ PTFV1 (mm·ms, post–pre)** | Mixed-effects, unadjusted | −1.4 | −1.7 – −1.1 | <0.001 |
|  | Mixed-effects, adjusted for LAVI | −1.3 | −1.6 – −1.0 | <0.001 |
| **AF recurrence (RF group)** | Cox, unadjusted PWD (per 10 ms) | HR 1.18 | 1.05 – 1.34 | 0.008 |
|  | Cox, +LAVI +AAD use | HR 1.17 | 1.04 – 1.32 | 0.011 |
| **AF recurrence (Cryo group)** | Cox, unadjusted PWD (per 10 ms) | HR 1.15 | 1.03 – 1.30 | 0.014 |
|  | Cox, +LAVI +AAD use | HR 1.14 | 1.02 – 1.29 | 0.018 |
| **AF recurrence (PFA group)** | Cox, unadjusted PWD (per 10 ms) | HR 1.14 | 1.01 – 1.31 | 0.036 |
|  | Cox, +LAVI +AAD use | HR 1.13 | 1.00 – 1.30 | 0.044 |

Mixed-effects models included patient-level random intercepts and were nested within the patient. Fixed effects: time (pre vs post), modality, lead, and interactions. Adjusted models included baseline indexed LA volume (mL/m²).
Cox models adjusted for age, sex, BMI, hypertension, indexed LA volume, and post-procedural AAD use (flecainide, sotalol). PWD scaled per 10 ms.

AF, atrial fibrillation; HR, hazard ratio; CI, confidence interval; RF, radiofrequency ablation; Cryo, cryoballoon ablation; PFA, pulsed-field ablation; PWD, corrected P-wave duration; PWDisp, P-wave dispersion; PWV, P-wave voltage; PTFV1, P-wave terminal force in lead V1; LAVI, left atrial volume index; AAD, anti-arrhythmic drug; LA, left atrial; BMI, body mass index; ms, milliseconds; mV·ms, millivolt-milliseconds.
